# Quetiapine-induced bicytopenia. Case report and literature review

**DOI:** 10.1192/j.eurpsy.2021.1282

**Published:** 2021-08-13

**Authors:** T. Gutiérrez Higueras, S. Vicent Forés, S. Sainz De La Cuesta Alonso, F. Calera Cortés

**Affiliations:** Clinical Unit Of Mental Health., Reina Sofia University Hospital., Córdoba., Spain

**Keywords:** Quetiapine, bycitopenia, antipsychotic, adverse reaction

## Abstract

**Introduction:**

Low white blood cell counts and agranulocytosis are a relatively rare side effect of atypical antipsychotic treatment. Like most atypical antipsychotics, quetiapine only has a 1%-4% risk of low blood cell count. The mechanism by which quetiapine causes these adverse effects is still unclear, some authors have proposed that this drug acts directly as a cytotoxic agent on immune cells and produces cell death, or the products of these drug could induce apoptosis by oxidative stress. Other authors have suggested a bone marrow depression, which could be produced by an inhibitory effect on leukopoiesis.

**Objectives:**

Presentation of a case of a bycitopenia after initiation of Quetiapine Prolong treatment to bipolar disorder and a literature review.

**Methods:**

We carried out a literature review in Pubmed electing those articles focused on cases of patients being treated with quetiapine and cytopenia as a side effect.

**Results:**

A 43-year-old woman with type I bipolar disorder is being treated with quetiapine prolong (50mg). After 6 years bicytopenia (anemia + leukopenia) was discovered in a routine analysis. In the Haematology Unit, long-term treatment with Quetiapine Prolong was found to be the cause of bicytopenia, having ruled out other ethological causes. This drug was suspended and switched to Aripiprazol. Eventually, the remission of symptoms and normalization of analytical parameters were achieved.

**Conclusions:**

In this case highlights the importance of understanding antipsychotic medications and their effects on the haematological system. Quetiapine Prolong produced bycitpoenia (anemia and thrombocytopenia), especially in long treatments. Therefore, clinical practitioners should be aware of this adverse effect.

